# An Anatomy-Informed Cross-Attention Framework for sEMG-Driven Knee and Ankle Moment Prediction During Sit-to-Walk Transitions

**DOI:** 10.3390/bioengineering13070798

**Published:** 2026-07-12

**Authors:** Jiarong Wu, Xinhao Wu, Qiuxia Zhang, Wanli Zang

**Affiliations:** School of Physical Education, Soochow University, Suzhou 215021, China

**Keywords:** sit-to-walk transition, surface electromyography (sEMG), inter-segmental cross-attention, bidirectional long short-term memory (BiLSTM), joint moment prediction

## Abstract

Sit-to-walk (STW) is a short-duration, high-load, multijoint transition requiring rapid lower-limb neuromuscular coordination across seat-off, load transfer, and gait initiation. Surface electromyography (sEMG)-based prediction of knee and ankle joint moments may support motor function evaluation and inform future assistive-control applications, but existing models remain limited in modeling cross-muscle sEMG feature interactions and mitigating phase-dependent prediction errors. This study developed an anatomy-informed framework for sEMG-driven moment prediction during STW. The model encoded sEMG channels from thigh and shank muscles into separate anatomical branches. Cross-Attention was used to model task-relevant intersegmental interactions, and BiLSTM was applied to capture short-term temporal dependencies. Eighteen healthy participants performed STW trials while sEMG, three-dimensional kinematics, and ground reaction forces were synchronously collected. Knee and ankle moments were calculated using inverse dynamics and used as reference targets. Among six models, the Cross-Attention model achieved the lowest test-set overall error, with an Overall nRMSE Fixed of 4.51%; the knee peak error in the P3 unloading phase was 16.17%. Ablation experiments indicated that Cross-Attention, BiLSTM temporal modeling, anatomical branch separation, and joint-specific output mapping contributed to prediction performance. This framework provides an interpretable approach for sEMG-driven multijoint moment prediction in complex non-stationary movements.

## 1. Introduction

Sit-to-walk (STW) is a common and representative functional transitional movement in daily life. Unlike simple sit-to-stand or steady-state walking, STW integrates forward trunk displacement, seat-off, load transfer, and gait initiation into a continuous action sequence, requiring the lower-limb neuromuscular system to rapidly transition from seated support to dynamic gait propulsion within a short period [1,2]. During STW, the knee and ankle joints play key roles in support regulation, lower-limb extension, load transfer, and propulsion control, and their joint moments reflect lower-limb mechanical loading and multijoint coordination characteristics [2,3]. Therefore, accurate assessment of knee and ankle joint moments during STW is important for understanding neuromuscular control strategies underlying functional movement transitions and for providing a reliable methodological basis for assist-as-needed (AAN) control in robotic rehabilitation systems and continuous motor function evaluation [4,5].

Currently, knee and ankle joint moments are typically obtained using three-dimensional motion capture, ground reaction force (GRF) measurements, and inverse dynamics. Although this approach provides high-precision biomechanical estimates, it requires laboratory-grade equipment, complex data processing, and subject-specific modeling, limiting its direct application in real-time rehabilitation assessment and assistive control scenarios [6]. Surface electromyography (sEMG) is a non-invasive signal that reflects neuromuscular activation during muscle contraction and provides indirect information about motor intent and neuromuscular control [7]. Previous studies have used sEMG for action recognition, continuous joint motion prediction, rehabilitation robot control, and human–machine interaction [8,9]. In parallel, data-driven biomechanical modeling has been increasingly used to estimate lower-limb kinematics, joint moments, and ground reaction forces from wearable or multimodal signals, providing a potential pathway toward more accessible movement assessment and assistive-control applications [10,11]. However, sEMG signals are susceptible to electrode placement, skin impedance, muscle fatigue, motion artifacts, and inter-subject variability, and are therefore highly nonstationary and nonlinear [8,12,13]. Moreover, compared with joint angles, the mapping from sEMG to joint moments involves complex muscle contraction dynamics and electromyography-to-mechanics conversion, making continuous sEMG-based joint moment prediction challenging [9,12].

Existing sEMG-driven biomechanical estimation models have evolved from conventional regression and machine-learning methods to deep learning architectures such as convolutional neural networks, recurrent architectures including LSTM/BiLSTM, and hybrid CNN–RNN frameworks [6,12,14,15]. Such deep learning models have improved the capacity to extract local temporal features and model sequential dependencies from multichannel sEMG signals. Building on these architectures, attention-based mechanisms have more recently been incorporated into EMG-based models to reweight informative temporal segments, muscle channels, or intermuscular relationships, and attention-enhanced recurrent or Transformer-based models have been explored for EMG-driven movement and biomechanical estimation [16,17,18]. Despite recent progress, sEMG-driven predictive models still face several limitations. First, many studies have focused on kinematic variables such as joint angles, whereas fewer have examined continuous prediction of kinetic variables such as knee and ankle joint moments. Unlike joint angles, joint moments directly reflect external loading, muscle mechanical output, and multijoint biomechanical control, making them important for both research and application [6,7,8]. Second, prior work has largely focused on fine upper-limb movements or steady-state walking, with limited attention to highly non-stationary tasks such as STW, which involves posture transition, seat-off, load transfer, and gait initiation [9,12]. Third, although some models incorporate multichannel sEMG, anatomical priors, or muscle synergy information, or attention mechanisms, many do not explicitly preserve anatomically separated thigh–shank feature streams before modeling inter-segmental interactions. These modeling approaches may be insufficient to capture phase-specific intermuscular coordination across anatomical segments, potentially limiting predictive robustness at dynamic transition points such as seat-off [13].

During STW, the thigh and shank muscles play distinct but interdependent roles in load transfer, support regulation, balance control, and propulsion, jointly supporting the transition from seated support to gait initiation [1,2]. Therefore, explicitly modeling cross-segment interactions based on anatomical segmentation may improve the accuracy and robustness of knee and ankle joint moment prediction during STW [18]. To address this gap, the present study proposes an anatomy-informed Cross-Attention CNN-BiLSTM framework. In this framework, sEMG channels are divided into thigh and shank branches to extract local spatiotemporal features; Cross-Attention is used to model task-relevant interactions between the two segments; and BiLSTM captures temporal dependencies during the movement transition. Unlike self-attention or a standard Transformer encoder, which primarily models dependencies within a unified feature sequence, the proposed Cross-Attention module was selected to preserve anatomically separated thigh–shank feature streams and enable directed information exchange between these two functionally coupled muscle groups. This design allows inter-segmental coordination to be modeled while retaining CNN and BiLSTM components for local spatiotemporal feature extraction and short-term temporal modeling. Thus, the key difference from existing attention-based EMG models is not the use of attention alone, but the combination of anatomical branch separation and directed cross-segment information exchange for multijoint moment prediction during STW. Single-segment, anatomical-separated, mismatched, and simple feature-fusion comparison models were constructed to isolate the contributions of input muscle range, anatomical correspondence, and inter-segmental interaction. We hypothesize that, compared with conventional fusion strategies, Cross-Attention may better capture cross-muscle interaction features during key STW phases and improve the stability of knee joint moment prediction during load transfer. This study aims to develop an interpretable sEMG-driven framework for multijoint moment prediction in complex functional movements.

## 2. Materials and Methods

**Figure 1:**
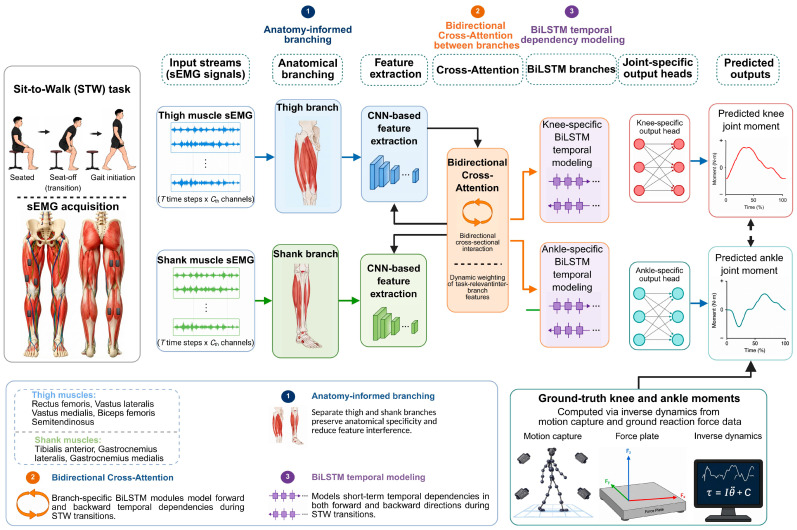
Overall workflow of the anatomy-informed Cross-Attention framework for sEMG-driven knee and ankle joint moment prediction during sit-to-walk transitions.

The overall workflow and architecture of the proposed anatomy-informed Cross-Attention framework are shown in Figure 1.

### 2.1. Subjects and Experimental Protocol

This study recruited 18 healthy adult participants (sex: 10 men and 8 women; age: 22.89 ± 2.54 years; body mass: 68.92 ± 11.68 kg; height: 173.1 ± 8.86 cm), none of whom had neurological disorders or recent lower-limb injuries. All participants provided written informed consent. The study was approved by the Ethics Committee of Soochow University (approval number: SUDA20251015H11) and was conducted in accordance with the principles of the Declaration of Helsinki [19].

STW task was designed based on the initial sit-to-stand and gait-initiation component of the timed up and go (TUG) test [20]. During the experiment, participants sat on a backless, armless chair with both hands placed on their thighs, and the hip and knee joints were initially flexed at approximately 90°. A representative sequence of the STW task is shown in Supplementary Figure S2. Because this study focused on the continuous lower-limb-driven transition after seat-off, the STW analysis window was defined as the interval from movement initiation to the completion of one full gait cycle, excluding the static seated phase [2]. Before formal data collection, participants completed approximately 10 min of warm-up and task familiarization. Three successful trials were recorded for each participant. No constraint was imposed on the leading leg at movement initiation; participants performed the STW movement according to their natural preference. Trials were subsequently classified according to the actual leading leg. Regardless of the leading leg, biomechanical analyses and all prediction targets were consistently referenced to the anatomical right lower limb.

### 2.2. Data Preprocessing and Phase Segmentation

Kinematic data were recorded using a motion capture system comprising 16 infrared cameras (Vicon MX13, Oxford Metrics, Oxford, UK) at a sampling frequency of 100 Hz, with 38 reflective markers (14 mm diameter) placed on anatomical landmarks. The marker set is illustrated in Supplementary Figure S1, and detailed marker descriptions and placement locations are provided in Supplementary Table S1. Ground reaction force (GRF) data were synchronously recorded using four floor-embedded Kistler three-dimensional force plates (model 9281, Kistler Instrumente AG, Winterthur, Switzerland) at 1000 Hz. GRF signals were processed using a zero-phase, second-order Butterworth low-pass filter with a cutoff frequency of 25 Hz [21]. All kinematic and kinetic data were processed in Visual3D. Joint centers were determined from the corresponding anatomical marker trajectories (e.g., the knee joint center was defined as the midpoint between RKNE and RKNM). Body segment inertial parameters were estimated according to the Dempster anthropometric model, and the human body was modeled as a linked rigid-body system in Visual3D [22]. Three-dimensional hip, knee, and ankle joint angles were calculated using an x–y–z Cardan rotation sequence, in which joint angles were defined as the orientation of the distal segment relative to the proximal segment in the corresponding local coordinate system. Net sagittal-plane joint moments of the anatomical right knee and ankle were computed using the Newton–Euler inverse dynamics algorithm implemented in Visual3D and normalized to body mass (Nm/kg). Kinematic signals were processed using a zero-phase, second-order Butterworth low-pass filter with a cutoff frequency of 6 Hz.

Surface electromyography (sEMG) signals were acquired from eight right lower-limb muscles using a wireless bipolar surface electrode system at a sampling frequency of 1000 Hz: biceps femoris (BF), lateral gastrocnemius (GL), medial gastrocnemius (GM), rectus femoris (RF), semitendinosus (ST), tibialis anterior (TA), vastus lateralis (VL), and vastus medialis (VM). Electrode placement followed SENIAM recommendations and was performed by trained personnel after palpation and fixation [23]. All devices were hardware-synchronized. Correspondingly, the prediction targets throughout this study were consistently defined as the sagittal-plane joint moments of the anatomical right ankle and right knee, irrespective of whether gait initiation was performed with the right or left leading leg. Thus, the classification of trials according to the leading leg was used only for subgroup analysis and did not alter the definition of the predicted joint moments. sEMG signals were processed using a zero-phase, fourth-order Butterworth band-pass filter (20–400 Hz) to remove motion artifacts and high-frequency noise; the filtered signals were then full-wave rectified and low-pass filtered at 6 Hz to obtain smooth linear envelopes [16,24]. For each STW trial, the sEMG envelope of each muscle was independently normalized to the maximum amplitude of the corresponding muscle within the same trial and scaled to a 0–1 range. The processed signals were then time-normalized to the STW cycle and resampled to 101 points (0–100% of the cycle).

To improve the robustness of phase identification, a multi-signal fusion strategy was used for event detection and cycle segmentation. This strategy integrated pelvic vertical displacement and velocity, bilateral lower-limb joint angles, and angular velocities, together with predefined kinematic criteria and duration constraints for automated segmentation. During the STW task, participants initially placed both feet on a single force plate, followed by sequential foot contact on two separate force plates after gait initiation. Only successful trials with complete foot contact on the designated force plates were included in the analysis; therefore, no manual force assignment or force redistribution was required in Visual3D. The STW cycle was divided into four functional phases: P1 (flexion phase), P2 (extension phase), P3 (unloading phase), and P4 (support phase). Specifically, P1 corresponds to trunk forward flexion and momentum generation; P2 corresponds to trunk and lower-limb extension during rising; P3 begins at Seat-off and corresponds to load transfer and swing-limb unloading; and P4 corresponds to the initial support and propulsion phase following gait initiation.

### 2.3. Models

Multiple CNN-BiLSTM prediction models were developed to evaluate the effects of input muscle range, anatomical correspondence, feature fusion, and inter-segmental interaction mechanisms [14,15,25]. For ease of description, RF, VL, VM, ST, and BF are collectively referred to as the thigh muscles, whereas TA, GL, and GM are collectively referred to as the shank muscles. Unless otherwise specified, all models used preprocessed sEMG signals as input and predicted knee and/or ankle joint moments. The core training procedure, hyperparameter settings, and model selection strategy are described in Section 2.3.7.

#### 2.3.1. Single-Segment Dual-Output CNN-BiLSTM Models (A1: Thigh-Only; A2: Shank-Only)

To evaluate the ability of sEMG signals from a single anatomical segment to predict knee and ankle joint moments, two single-segment dual-output CNN-BiLSTM models were constructed [14,15,25]. The thigh-only model (A1) used only thigh muscle signals, whereas the shank-only model (A2) used only shank muscle signals. Both models simultaneously predicted ankle and knee joint moments. No cross-segmental information was incorporated, so these models served as baselines for dual-joint moment prediction using a single muscle segment.

#### 2.3.2. Anatomical-Separated CNN-BiLSTM Model (B: Anatomical-Separated)

Based on the anatomical correspondence between lower-limb muscles and joint motion, an anatomical-separated CNN-BiLSTM model was constructed [18,24]. This model separately modeled the thigh and shank muscles, with the thigh branch predicting the knee joint moment and the shank branch predicting the ankle joint moment. No feature fusion or information transfer was performed between the two branches. For consistent comparison with dual-output models, the two prediction outputs were organized in the order “ankle joint–knee joint.”

#### 2.3.3. Mismatched-Separated CNN-BiLSTM Model (C: Mismatched-Separated)

To construct a negative control for anatomical correspondence, a mismatched-separated CNN-BiLSTM model was designed. This model retained the separated modeling framework but intentionally reversed the correspondence between input muscle groups and prediction targets. Specifically, thigh muscle signals were used to predict the ankle joint moment, whereas shank muscle signals were used to predict the knee joint moment. The network architecture was otherwise identical to the anatomical-separated model, thereby forming an anatomical-mismatch negative control.

#### 2.3.4. Feature-Fusion CNN-BiLSTM Model (D: Feature-Fusion)

Based on the separated modeling framework, a feature-fusion CNN-BiLSTM model was constructed to simultaneously utilize thigh and shank muscle signals [14,25]. This model included two CNN encoding branches, one for each anatomical segment. The extracted thigh and shank features were concatenated along the feature dimension to form a unified dual-segment representation, which was then input into a shared BiLSTM and mapped to ankle and knee joint moments. This model achieved dual-segment fusion through direct feature concatenation, without explicit inter-segmental cross-attention.

#### 2.3.5. Inter-Segmental Cross-Attention CNN-BiLSTM Model (E: Cross-Attention)

Based on the feature-fusion model, this study further constructed an inter-segmental CNN-BiLSTM model to explicitly capture task-relevant interactions between thigh and shank sEMG features [18,26,27]. The model used dual-branch inputs consisting of thigh muscles (RF, VL, VM, ST, and BF) and shank muscles (TA, GL, and GM), and simultaneously predicted knee and ankle joint moments.

Two CNN encoders separately extracted local spatiotemporal features from the two anatomical branches and mapped them into a shared feature space. Bidirectional Cross-Attention was then applied for inter-segmental information exchange. Specifically, the knee prediction branch used thigh features as the Query to retrieve synergistic information from shank features, whereas the ankle prediction branch used shank features as the Query to extract support- and propulsion-related information from thigh features. The resulting interaction-enhanced features were then input into the corresponding BiLSTM branches for temporal modeling and passed through fully connected layers to output knee and ankle joint moments.

Compared with direct feature concatenation, Cross-Attention allows one anatomical branch to selectively retrieve task-relevant information from the other branch while preserving segment-specific representations. This design was chosen because the present task involved two anatomically separated but functionally coupled muscle groups. In contrast, self-attention or a standard Transformer encoder applied after concatenating the two feature streams would mainly model dependencies within a unified sequence, which may obscure the explicit anatomical separation between thigh and shank muscles. Therefore, Cross-Attention was considered more appropriate for modeling directed thigh–shank interactions during STW. Because CNN and BiLSTM modules were already used for local spatiotemporal feature extraction and short-term temporal modeling, respectively, a full Transformer encoder was not introduced to avoid additional model complexity and potential overfitting risk in a relatively small-sample biomechanical dataset. Cross-Attention weights were retained during testing for subsequent intermuscular interaction analysis (see Figure 1).

#### 2.3.6. Ablation Experiments

To evaluate the contribution of different structural modules to knee and ankle joint moment prediction, ablation models were constructed based on the full E: Cross-Attention model. Except for the modules that were replaced or removed, all ablation models used identical data preprocessing, sliding-window settings, input–output formats, and training strategies to ensure comparability.

The dual-branch CNN-BiLSTM model removed the Cross-Attention module between the thigh and shank branches and directly concatenated the two feature sets before BiLSTM-based temporal modeling. The one-way Cross-Attention model retained only one direction of inter-segmental attention, such that one joint prediction branch received information from the other segment whereas the reverse interaction was removed. The shared output head model retained the dual-branch CNN encoders and bidirectional Cross-Attention module but replaced the independent knee and ankle prediction heads with a shared BiLSTM and fully connected output head.

The single-branch Cross-Attention model removed anatomical branch separation and input all sEMG channels into a single CNN encoder before constructing knee and ankle prediction branches for Cross-Attention interaction. The Cross-Attention-MLP model retained the dual-branch CNN encoders and bidirectional Cross-Attention module but replaced the BiLSTM temporal modeling layer with an MLP. These comparisons were used to evaluate the contributions of Cross-Attention, anatomical branch separation, independent prediction heads, and BiLSTM temporal modeling to overall model performance.

**Figure 2:**
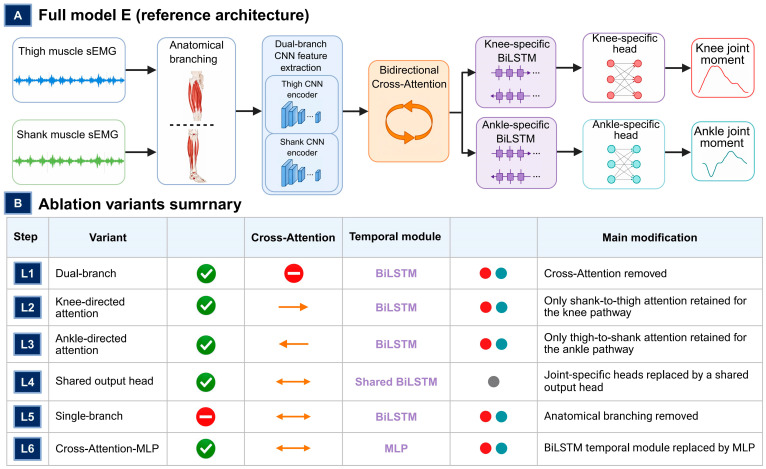
Reference architecture of the full Cross-Attention model and summary of ablation variants.

The full reference architecture and the corresponding ablation variants are summarized in Figure 2.

#### 2.3.7. Hyperparameter Settings and Model Training

To prevent information leakage, dataset partitioning was performed at the participant level. Participants were randomly divided into a training-validation set (80%) and an independent test set (20%). The training-validation set was further randomly split into training (80%) and validation (20%) subsets for hyperparameter optimization. Consequently, all trials and derived overlapping sliding windows from the same participant were assigned exclusively to a single subset and were never shared across the training, validation, and test sets during model training and evaluation.

To maintain consistency with the kinematic and kinetic data, sEMG linear envelopes were resampled to 100 Hz before model input. The preprocessed sEMG signals were segmented into 200 ms windows, each containing 20 samples with 80% overlap, corresponding to a step size of 4 samples. Ten consecutive windows were combined to form one input sequence for characterizing short-term sEMG dynamics [16,24].

For the network architecture, CNN modules used 3 × 3 convolution kernels, together with batch normalization, LeakyReLU activation, average pooling, and Dropout for feature extraction and regularization. Downsampling was applied mainly along the temporal dimension to preserve the muscle-channel dimension. BiLSTM modules captured temporal dependencies across consecutive windows, and fully connected layers performed joint moment regression.

During training, all models used the AdamW optimizer with weight decay, learning-rate scheduling, gradient clipping, and early stopping to improve training stability [28]. Dual-output models employed a weighted mean squared error for knee and ankle tasks, whereas single-output models used single-target mean squared error. Key hyperparameters—including learning rate, weight decay coefficient, Dropout ratio, number of convolutional channels, BiLSTM hidden dimension, batch size, and loss-function weights—were determined using the inner validation set.

Despite differences in input channel combinations, output forms, and feature interactions across experiments, the training strategy, optimization criteria, and model selection standards remained consistent to ensure fair model comparison. To ensure clarity and reproducibility, the detailed network configurations, including the number of convolutional layers, channel sizes, kernel settings, training epochs, random seed, and model selection criteria, are provided in Supplementary Table S2.

All models were implemented in PyTorch 2.8.0 and trained using Python 3.11 with CUDA 12.8 on Windows 10 Professional 22H2. Training was conducted on an NVIDIA GeForce RTX 5090 GPU, supported by an Intel Core Ultra 9 285K CPU and 128 GB of RAM.

#### 2.3.8. Performance Metrics

Because this study focused on comparing prediction errors for knee and ankle joint moments across models, error-based metrics were used as the primary performance measures. These included root mean square error (RMSE), mean absolute error (MAE), and fixed-range normalized root mean square error (nRMSE Fixed) [9]. RMSE quantifies the overall magnitude of deviation between predicted and true values, MAE reflects the average absolute error, and nRMSE Fixed standardizes RMSE using a predefined fixed normalization range to reduce the influence of amplitude differences across test sets, thereby enhancing comparability across models.

The definitions of each metric are as follows:

$$\mathrm{RMSE}=\sqrt{\frac{1}{N}\sum_{i=1}^{N}{\left(\theta_{i}-{\hat{\theta }}_{i}\right)}^{2}}$$

$$\mathrm{MAE}=\frac{1}{N}\sum_{i=1}^{N}\left|\theta_{i}-{\hat{\theta }}_{i}\right|$$

$$\mathrm{nRMS}E_{\mathrm{fixed}}=\frac{\mathrm{RMSE}}{R_{\mathrm{fixed}}}\times 100\%$$

where $\theta_{i}$ and ${\hat{\theta }}_{i}$ denote the ground-truth and predicted values of the i-th sample, respectively, and N represents the total number of samples. $R_{\mathrm{fixed}}$ is a predefined fixed normalization range. For the dual-output model, ankle and knee joint moment errors were computed separately, and their mean value was further used as the overall performance metric.

### 2.4. Statistical Analysis

In descriptive analyses and visualizations, individual trials were treated as the basic analytical unit, whereas statistical inference was performed at the subject level to ensure independence of observations. For each trial within the corresponding dataset subset, Overall, Ankle, and Knee nRMSE Fixed metrics were extracted. Overall was defined as the arithmetic mean of the ankle and knee nRMSE Fixed values. For statistical comparisons, trial-level metrics were first aggregated within each participant for each model, dataset subset, and outcome metric, and the resulting subject-level values were used as the independent analytical units. Model results were summarized using the mean, standard deviation, and median, and subject-level distribution plots were used for visualization.

Because subject-level aggregated values were paired across models, differences between models were analyzed using the paired Wilcoxon signed-rank test. For the six main-model comparison, the E: Cross-Attention model served as the reference, and pairwise comparisons were performed against each of the other five baseline models. Analyses were conducted separately for the training and test sets and independently for the Overall, Ankle, and Knee metrics. Multiple comparisons were corrected using the Holm–Bonferroni method, with the significance level set at α = 0.05. Results from the independent test set were used to evaluate model generalization, whereas training set results served as a reference for fit stability during training.

## 3. Results

### 3.1. Phase-Specific Lower-Limb Neuromuscular and Biomechanical Features

**Figure 3:**
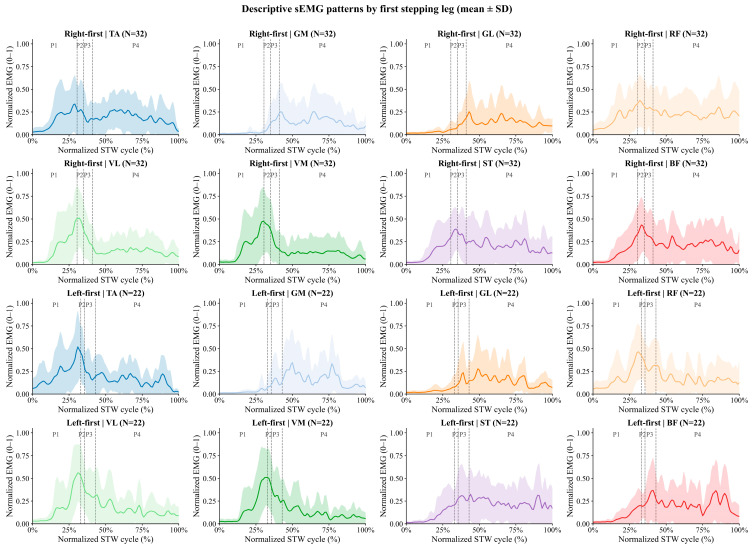
Lower-limb sEMG activation patterns during the sit-to-walk transition according to the leading leg. Normalized sEMG envelopes of eight lower-limb muscles are shown across the normalized STW cycle (0–100%) for trials initiated with the right and left leading legs. Solid lines denote group means, shaded areas indicate standard deviations, and vertical dashed lines mark the boundaries of the four functional phases. P1–P4 represent the flexion, extension, unloading, and support phases, respectively.

**Figure 4:**
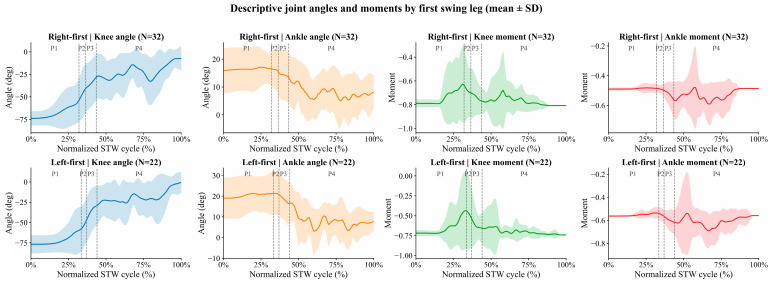
Knee and ankle joint angle and moment profiles during the sit-to-walk transition according to the leading leg. Swing-limb knee and ankle joint angles and moments are shown across the normalized STW cycle (0–100%) for trials initiated with the right and left leading legs. Solid lines denote group means, shaded areas indicate standard deviations, and vertical dashed lines mark the boundaries of the four functional phases. P1–P4 represent the flexion, extension, unloading, and support phases, respectively.

Valid trials were divided into a right-leading-leg group (*n* = 32) and a left-leading-leg group (*n* = 22). Figure 3 and Figure 4 show the average normalized sEMG envelopes of eight right lower-limb muscles, together with knee and ankle joint angles and moments across the STW cycle (0–100%) under the two leading-leg conditions. In the right-leading-leg group, the vastus lateralis (VL) and vastus medialis (VM) showed higher average peak activations of 0.509 and 0.474, respectively, whereas the medial gastrocnemius (GM) and lateral gastrocnemius (GL) showed lower peaks of 0.255 and 0.253, respectively. In this group, knee and ankle joint angles ranged from −73.847° to −7.332° and from 5.002° to 17.133°, respectively. Knee and ankle joint moments ranged from −0.808 to −0.628 and from −0.592 to −0.479, respectively. In the left-leading-leg group, VL, tibialis anterior (TA), and VM showed higher average peak activations of 0.560, 0.520, and 0.512, respectively, whereas GL remained relatively low at 0.279. Knee and ankle joint angles ranged from −76.682° to −0.801° and from 3.116° to 21.385°, respectively. Knee and ankle joint moments ranged from −0.740 to −0.435 and from −0.690 to −0.536, respectively.

### 3.2. Model Prediction Performance

#### 3.2.1. Overall and Joint-Specific Results on the Test Set

**Figure 5:**
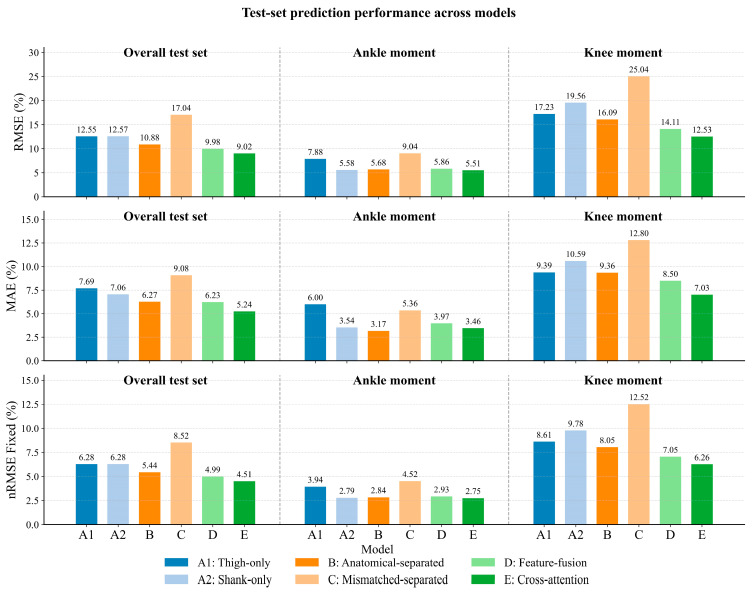
Test-set prediction performance of the six models for knee and ankle joint moment estimation.

Figure 5 presents the test-set prediction performance of the six models at the Overall, Ankle, and Knee levels using RMSE, MAE, and nRMSE Fixed. For visualization, RMSE and MAE are expressed as percentages in the figure, while nRMSE Fixed retains its original percentage values.

At the Overall level, the E: Cross-Attention model showed the lowest errors among the six models, with RMSE, MAE, and nRMSE Fixed values of 9.02%, 5.24%, and 4.51%, respectively. Compared with D: Feature-fusion and B: Anatomical-separated, the E model reduced Overall nRMSE Fixed by 9.67% and 17.15%, respectively.

At the joint-specific level, the E: Cross-Attention model showed the lowest Ankle RMSE and Ankle nRMSE Fixed, as well as the lowest RMSE, MAE, and nRMSE Fixed for the Knee. The Ankle nRMSE Fixed was 2.75%, and the Knee nRMSE Fixed was 6.26%. The A2: Shank-only model reached an Ankle nRMSE Fixed of 3.00%, close to that of the E model, but showed 11.22–22.16% higher errors across all Knee metrics. The C: Mismatched-separated model had the highest errors at both Overall and joint-specific levels. B: Anatomical-separated showed lower Overall errors than C, whereas D: Feature-fusion showed slightly lower Overall errors than B. Taken together, the E: Cross-Attention model achieved the lowest mean errors across most metrics.

#### 3.2.2. Individual Subject Prediction Performance

**Figure 6:**
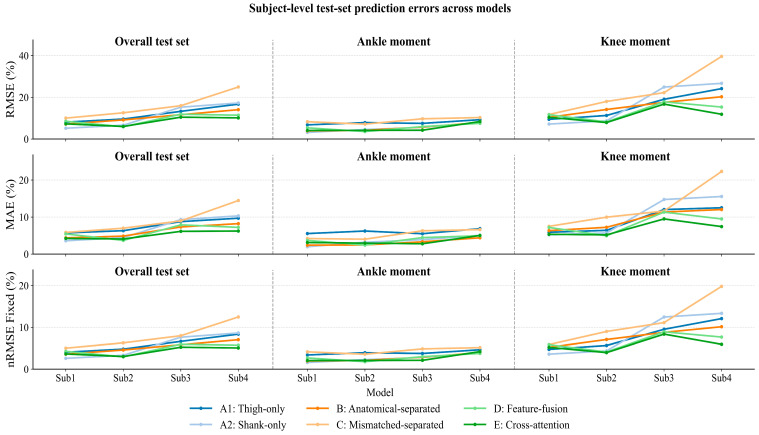
Subject-level prediction error comparison among the six models on the test set.

Figure 6 presents prediction performance at the individual subject level. The E: Cross-Attention model showed lower errors for most test subjects and maintained comparatively low errors even in subjects 3 and 4, who showed relatively higher errors across models. This indicates good individual-level stability. Joint-specific results showed that the advantage of the E: Cross-Attention model was more pronounced for Knee prediction. For example, subject 4 had a Knee nRMSE Fixed of 5.94%, lower than 7.66% for D: Feature-fusion and 10.13% for B: Anatomical-separated.

### 3.3. Subject-Level Prediction Error Distribution

**Figure 7:**
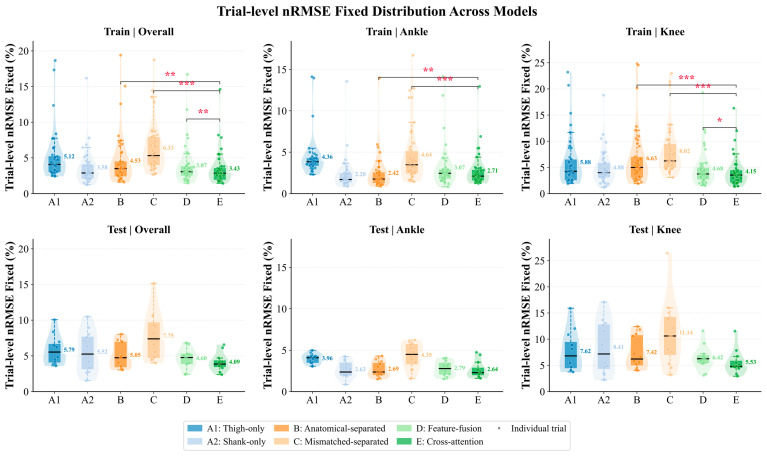
Subject-level nRMSE Fixed distributions and statistical comparisons across the six models. Trial-level errors were first averaged within each participant, and the resulting subject-level values are shown separately for the training and test sets. Pairwise comparisons were performed at the subject level using the E: Cross-Attention model as the reference. Significance annotations are based on Holm–Bonferroni-adjusted *p*-values. * *p* < 0.05, ** *p* < 0.01, and *** *p* < 0.001.

Figure 7 presents the subject-level nRMSE Fixed distributions of the six models for both the training and test sets. Paired Wilcoxon signed-rank tests were conducted at the subject level using E: Cross-Attention as the reference model, with Holm–Bonferroni correction applied for multiple comparisons.

In the training set, E: Cross-Attention achieved the lowest mean nRMSE Fixed values for Overall (3.43%) and Knee (4.15%) predictions among all models. Statistically significant differences were observed for Overall performance between E and D: Feature-fusion (*p* = 0.0046), B: Anatomical-separated (*p* = 0.0011), and C: Mismatched-separated (*p* < 0.001). Similar significant improvements were also observed for Knee prediction when comparing E with D, B, C, and A1 (all *p* < 0.05). For Ankle prediction, E significantly outperformed A1, A2, B, and C (*p* < 0.05), while no significant difference was observed between E and D.

In the test set, although E: Cross-Attention consistently showed the lowest mean values across models (Overall: 4.09%, Knee: 5.53%, Ankle: 2.64%), none of the pairwise comparisons reached statistical significance after Holm–Bonferroni correction (all adjusted *p* > 0.05).

### 3.4. Error Distribution Across the Action Cycle

**Figure 8:**
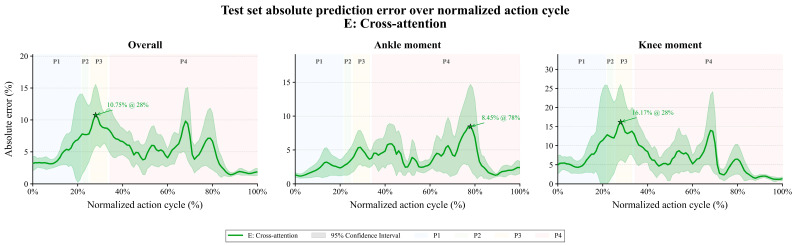
Absolute prediction error of the E: Cross-Attention model across the normalized sit-to-walk cycle. Absolute prediction errors of the E: Cross-Attention model are shown across the normalized STW cycle in the independent test set. Error curves are presented at the Overall, Ankle, and Knee levels. Vertical dashed lines indicate the boundaries of the four functional phases. P1–P4 represent the flexion, extension, unloading, and support phases, respectively.

Figure 8 shows the absolute prediction error distribution of the E: Cross-Attention model across the STW cycle in the test set. The overall MAE peak occurred at approximately 28% of the cycle, corresponding to P3: unloading phase, with a value of 10.75%. At the joint level, Knee error also peaked during P3 at 16.17%, whereas Ankle error peaked at 8.45% around 78% of the cycle, corresponding to the late portion of P4: support phase. Knee errors were primarily concentrated during load transfer and swing-limb unloading, whereas Ankle errors were more prominent during post-support propulsion.

Across models, Knee and Overall peak errors were generally concentrated in P3. A1, A2, B, D, and E showed Overall peaks near 28% of the cycle, whereas C peaked near 27% (Supplementary Figures S3–S7). The E model had a lower overall peak error (10.75%) than D: Feature-fusion (12.94%), B: Anatomical-separated (16.19%), and C: Mismatched-separated (18.97%). Its Knee peak error was also lower than those of D, B, and C (16.17% vs. 22.03%, 28.43%, and 34.26%, respectively). Ankle peak errors occurred mostly during P4, with E (8.45%) close to D (7.53%) and A2 (7.67%), but lower than C (14.09%).

### 3.5. Ablation Study

Table 1 summarizes the test-set prediction performance of the full E: Cross-Attention model and six ablation variants. The complete E model showed the best overall balance across metrics, with an Overall nRMSE Fixed of 4.51%, Overall RMSE of 9.02%, and Overall MAE of 5.24%. Removing Cross-Attention increased Overall nRMSE Fixed from 4.51% to 5.53% in L1: Dual-branch without Cross-Attention, while Knee nRMSE Fixed increased from 6.26% to 8.30%. Among the one-way Cross-Attention ablations, L2: Knee-directed one-way Cross-Attention showed an Overall nRMSE Fixed of 4.90% and a Knee nRMSE Fixed of 6.86%. L3: Ankle-directed one-way Cross-Attention showed an Overall nRMSE Fixed of 4.48%, slightly lower than that of the full model, but its Overall MAE increased to 5.63%, compared with 5.24% for the full model.

The shared output head model, L4, had an Overall nRMSE Fixed of 5.25% and a Knee nRMSE Fixed of 7.62%, both higher than those of the full model. Removing anatomical branch separation resulted in L5, with an Overall nRMSE Fixed of 5.39% and a Knee nRMSE Fixed of 8.04%. Replacing the BiLSTM temporal modeling module with MLP resulted in L6, which showed an Overall nRMSE Fixed of 6.00% and an Overall MAE of 8.22%. Its Ankle nRMSE Fixed increased from 2.75% in the full model to 4.39%.

## 4. Discussion

In the STW task, the E: Cross-Attention model showed lower overall error and better knee joint moment prediction performance than the five baseline models, while maintaining low errors for ankle joint moment prediction. In particular, during P3: unloading phase, the knee peak error decreased from 22–34% in the other main baseline models to 16.17%, indicating better predictive performance during this highly dynamic load-transfer phase. Trial-level and individual-level results further showed that the E model maintained lower errors in most trials and participants. The ablation experiments indicated that Cross-Attention, BiLSTM, anatomical branch separation, and joint-specific output mapping all made important contributions to model performance. Overall, these results support the core hypothesis of this study: combining anatomical branches, inter-segmental cross-attention, and bidirectional temporal modeling may help improve the accuracy and stability of sEMG-driven knee and ankle joint moment prediction in non-stationary functional movements such as STW.

The Cross-Attention model showed clear phase-dependent prediction errors across the action cycle. The overall mean absolute error peak was approximately 10.75%, while the knee error peak (16.17%) was concentrated at approximately 28% of the action cycle, corresponding to P3: unloading phase; the ankle error peak occurred in the late portion of P4: support phase (8.45%). This error distribution was closely related to the biomechanical characteristics of STW. During P3: unloading phase, because of the forward-upward movement of the center of mass and the demand for load transfer, the quadriceps and other lower-limb extensors must rapidly contribute to support and extension control, thereby controlling center-of-mass movement at Seat-off and maintaining movement stability [2,3]. The ankle error peak occurring at approximately 78% of the action cycle, corresponding to the late portion of P4: support phase, may reflect the gradual transition from functional transfer to post-gait-initiation support and propulsion. At this stage, the ankle needs to contribute to support, propulsion, and dynamic balance regulation, and the recorded medial and lateral gastrocnemius signals may provide partial information related to plantarflexor-associated support and propulsion. These task demands may increase the non-stationarity of the sEMG–moment mapping and the difficulty of modeling this phase [3,29].

The ablation experiments further evaluated the contributions of each core component to knee and ankle joint moment prediction performance. After the Cross-Attention module was removed, overall and knee prediction performance decreased, suggesting that the contribution of inter-segmental muscle feature interaction to model performance was joint-specific, mainly reflected in knee joint moment prediction, whereas ankle joint moment prediction was less affected. The one-way Cross-Attention results further indicated that information flow in a single direction could preserve part of the predictive capability; however, the complete bidirectional structure showed more stable performance in Overall MAE, knee error, and multi-metric balance. Therefore, the advantage of bidirectional Cross-Attention was mainly reflected in a balanced benefit across multiple metrics, rather than absolute superiority in every single metric. Previous studies have also shown that attention mechanisms can facilitate complex sEMG spatiotemporal feature fusion, and that muscle synergy and anatomical prior modeling may improve continuous joint prediction performance [16,17,18]. This knee–ankle difference may reflect task specificity and anatomical locality in lower-limb joint moment generation. STW is a multi-stage transition task involving sitting, transfer, and gait initiation; therefore, knee joint moment prediction during load transfer may depend more strongly on cross-segmental coupling between thigh support muscles and shank coordination muscles. In contrast, ankle joint prediction in the late P4 phase is more closely related to propulsion-related control, and its predictive information may be mainly concentrated in distal shank muscles; accordingly, ankle prediction performance was less affected when cross-segmental interaction was absent [30,31].

Anatomical branch separation, joint-specific prediction heads, and BiLSTM-based temporal modeling also had important effects on model performance. After anatomical branch separation was removed or a shared output head was used, both overall error and knee error increased, indicating that anatomical priors and joint-specific output mapping may help reduce irrelevant feature interference and preserve the specificity of different joint prediction tasks. Anatomically partitioning the thigh and shank muscles may help retain the structural relationship between muscle groups and joint moments, whereas joint-specific prediction heads may separately model the different kinetic output patterns of the knee and ankle joints [16,18]. In addition, after BiLSTM was removed or replaced with MLP, overall prediction performance decreased, with clear increases in both Overall nRMSE Fixed and Overall MAE, indicating that bidirectional temporal modeling may help represent dynamic dependencies during movement transitions. Cross-Attention can enhance spatial interaction modeling between muscle groups, but without temporal dependence modeling across the action cycle, the model may still have difficulty stably representing the rapidly changing kinetic features of load transfer, Seat-off, and propulsion phases. Previous studies on lower-limb joint moment prediction have also indicated that LSTM-based models can be used to model temporal dependence in continuous kinetic prediction [32,33]. Overall, the performance improvement of the E: Cross-Attention model was not attributable to a single structure alone, but rather to the combined effects of inter-segmental feature interaction, bidirectional temporal modeling, anatomical branch separation, and joint-specific output mapping.

The anatomical branch design based on physiological priors further enhanced the biomechanical interpretability of the model representation. Unlike unstructured concatenation of all sEMG channels, the anatomical branches organized input features according to the functional regions of the thigh and shank muscles, allowing the model to structurally retain the anatomical correspondence between muscle groups and joint moments. Subsequently, the Cross-Attention mechanism allowed task-relevant feature interactions between different anatomical branches, enabling the knee and ankle prediction branches to integrate complementary features from the other anatomical region. This design is consistent with muscle synergy theory, in which complex movements are not controlled by a single muscle in isolation but are achieved through coordinated activation across multiple muscle groups [30,31]. Previous sEMG studies have also shown that incorporating muscle anatomy, electrode placement, or muscle synergy information into feature extraction can help obtain more effective representations of muscle function and improve continuous joint motion prediction performance [16,18]. Therefore, the combination of anatomical branches and Cross-Attention provides a more biomechanically grounded model representation for cross-regional sEMG feature modeling in STW. The model prediction performance and neuromuscular mechanism interpretation should be considered separately: the former reflects the accuracy of data-driven sEMG-to-moment mapping, whereas the latter provides only indirect and exploratory clues about intermuscular coordination. Furthermore, this strategy provides a scalable approach for lower-limb joint moment prediction in complex non-stationary movements and may be applied in the future to continuous moment estimation, movement intention recognition, rehabilitation robotics, and assist-as-needed control of exoskeletons.

A direct quantitative comparison with externally reported state-of-the-art EMG/sEMG-based biomechanical estimation results should be interpreted cautiously because previous studies differ substantially in movement tasks, input modalities, target variables, normalization procedures, data-splitting strategies, and evaluation metrics. Therefore, numerical cross-study comparison may be misleading. Instead, this study emphasized controlled within-dataset comparisons under identical preprocessing, training, and evaluation conditions. Comparisons with single-segment, anatomical-separated, mismatched, feature-fusion, and ablation models allowed the contributions of anatomical correspondence, inter-segmental Cross-Attention, BiLSTM temporal modeling, and joint-specific output mapping to be evaluated more fairly. Thus, the present results should be interpreted as evidence supporting the methodological value of the proposed anatomy-informed Cross-Attention framework during STW rather than as a direct superiority claim over all existing methods. Recent studies on finite-element-based lattice insole optimization further demonstrate the broader value of machine learning in biomechanical modeling and device optimization [34].

This study has several limitations. First, the experimental sample consisted primarily of healthy young adults, and the applicability of the model to older adults or populations with impaired motor function, such as individuals with stroke, lower-limb injuries, or neuromuscular control deficits, remains unclear. Therefore, future studies should include more heterogeneous samples to evaluate its cross-population generalizability. Second, data collection relied on a laboratory-based Vicon system and embedded force plates, and model performance in wearable sensor settings or real-world scenarios may be affected by sensor noise, soft tissue artifacts, signal drift, and synchronization errors across multiple devices. In addition, all signals were time-normalized to the complete STW cycle before model input; therefore, the present framework should be interpreted as an offline prediction and methodological feasibility study rather than a fully real-time control system. Third, although Cross-Attention weights provide clues for interpreting information exchange between muscle groups, they primarily reflect statistical associations learned by a data-driven model and should be interpreted cautiously in terms of neurophysiological significance. Future work could combine high-density electromyography, motor unit discharge characteristics, or brain–muscle coupling analysis to further examine the potential relationship between model weights and neuromuscular control mechanisms. Fourth, this study focused on a standard STW task and did not cover variations in chair height, external loading, fatigue states, assistive device use, abnormal gait, or pathological movement patterns; therefore, model performance under these task and population conditions remains to be evaluated. Fifth, this study did not include a direct external benchmark comparison with state-of-the-art EMG/sEMG-based biomechanical estimation models. Future studies should evaluate the proposed framework using larger datasets, public datasets, shared benchmark datasets, or standardized experimental protocols to enable more direct comparison with existing methods. Finally, the proposed framework includes multiple architectural components, including anatomical branch separation, bidirectional Cross-Attention, BiLSTM temporal modeling, and joint-specific output heads. Future studies should further evaluate its model size, inference efficiency, and real-time deployment feasibility.

## 5. Conclusions

This study proposed an anatomy-informed sEMG-driven framework integrating Cross-Attention and BiLSTM for continuous prediction of knee and ankle joint moments during STW. The E: Cross-Attention model showed lower mean errors than the baseline models and reduced knee peak error during P3: unloading phase, while showing stable performance across trials and subjects. Ablation experiments indicated that inter-segmental feature interaction, bidirectional temporal modeling, anatomical branch separation, and joint-specific output mapping all made important contributions to predictive performance. By preserving lower-limb muscle anatomical information and modeling task-relevant thigh–shank interactions, the model improves the interpretability of sEMG-driven dynamic prediction in complex non-stationary functional movements. Future studies should further validate this approach in clinical populations, wearable sensor environments, and real-time control scenarios.

## Figures and Tables

**Table 1:** Test-set prediction performance of the full model and ablation variants.

Model Name	Ablated Component	Overall RMSE (%)	Overall MAE (%)	Overall nRMSE Fixed (%)	Ankle RMSE (%)	Ankle MAE (%)	Ankle nRMSE Fixed (%)	Knee RMSE (%)	Knee MAE (%)	Knee nRMSE Fixed (%)
Full model E	None	9.02	5.24	4.51	5.51	3.46	2.75	12.53	7.03	6.26
L1: Dual-branch without Cross-Attention model	Cross-Attention removed	11.05	6.25	5.53	5.52	3.49	2.76	16.59	9	8.3
L2: Knee-directed one-way Cross-Attention	Ankle-directed attention removed	9.79	5.76	4.9	5.87	3.4	2.94	13.72	8.13	6.86
L3: Ankle-directed one-way Cross-Attention	Knee-directed attention removed	8.96	5.63	4.48	5.47	3.84	2.73	12.45	7.42	6.23
L4: Shared output head	Joint-specific output heads replaced by a shared output head	10.5	6.2	5.25	5.77	3.9	2.89	15.23	8.5	7.62
L5: Single-branch Cross-Attention	Anatomical branch separation removed	10.78	6.2	5.39	5.49	3.83	2.74	16.07	8.57	8.04
L6: Cross-Attention-MLP	BiLSTM temporal module replaced by MLP	11.99	8.22	6	8.78	7.38	4.39	15.21	9.06	7.6

## Data Availability

The data presented in this study are available on reasonable request from the corresponding authors. The data are not publicly available due to privacy and ethical restrictions related to the human participant data.

## Supplementary Materials

The following supporting information can be downloaded at: https://www.mdpi.com/article/10.3390/bioengineering13070798/s1, Figure S1: Illustration of marker-set; Figure S2: Representative sequence of the sit-to-walk task; Figure S3: Absolute prediction error of the A1: Thigh-only model over the normalized action cycle in the test set; Figure S4: Absolute prediction error of the A2: Shank-only model over the normalized action cycle in the test set; Figure S5: Absolute prediction error of the B: Anatomical-separated model over the normalized action cycle in the test set; Figure S6: Absolute prediction error of the C: Mismatched-separated model over the normalized action cycle in the test set; Figure S7: Absolute prediction error of the D: Feature-fusion model over the normalized action cycle in the test set; Table S1: Description of markers and placement; Table S2: Common training configuration used for all models.

## Abbreviations

The following abbreviations are used in this manuscript:

**Abbreviation**	**Full term**
**AAN**	Assist-as-needed
**BF**	Biceps femoris
**BiLSTM**	Bidirectional long short-term memory
**CNN**	Convolutional neural network
**GL**	Lateral gastrocnemius
**GM**	Medial gastrocnemius
**GRF**	Ground reaction force
**MAE**	Mean absolute error
**MLP**	Multilayer perceptron
**nRMSE Fixed**	Fixed-range normalized root mean square error
**RF**	Rectus femoris
**RMSE**	Root mean square error
**sEMG**	Surface electromyography
**SENIAM**	Surface Electromyography for the Non-Invasive Assessment of Muscles
**ST**	Semitendinosus
**STW**	Sit-to-walk
**TA**	Tibialis anterior
**TUG**	Timed up and go
**VL**	Vastus lateralis
**VM**	Vastus medialis
