# Supplementary material for: An Anatomy-Informed Cross-Attention Framework for sEMG-Driven Knee and Ankle Moment Prediction During Sit-to-Walk Transitions
Source: Bioengineering (Basel). 2026 Jul 12;13(7):798. doi: 10.3390/bioengineering13070798 (PMC13405828; doi:10.3390/bioengineering13070798)
Supplement: Supplementary file 1 [file bioengineering-13-00798-s001.zip › bioengineering-4409343-supplementary.pdf]

Table S1. Description of markers and placement

No.	Labels		MARKER PLACEMENT
1	Head		Placed on the vertex of the head
2、3	Shoulder2	Shoulder1	Placed on the acromio-clavicular joint
4	Thorax		Spinous process of the 10th thoracic vertebrae
5、6	LASI	RASI	Placed directly over the left/right anterior superior iliac spine
7、8	LPSI	RPSI	Placed directly over the left/right posterior superior iliac spine
9、10	LTHI	RTHI	Place marker half way down the lateral lower leg in such a way that it and the hip and knee joint centers lie in the coronal plan of the femur. Position asymmetrically (in terms of height), left versus right
11、12	LTHAP	RTHAP	Place the marker one-third of the way down the center of the anterior thigh (use the ASIS marker and the epicondyle markers to define the length of the femur in this instance)
13、14	LTHAD	RTHAD	Place the marker two-thirds of the way down the center of the anterior thigh (use the ASIS marker and the epicondyle markers to define the length of the femur in this instance)
15、16	LKNE	RKNE	Placed on the lateral epicondyle of the knee
17、18	LKNM	RKNM	Placed on the medial femoral epicondyle
19、20	LTIB	RTIB	Place marker half way down the lateral lower leg in such a way that it and the knee and ankle joint centres lie in the coronal plane of the tibia. Position asymmetrically (in terms of height), left versus right
21、22	LTIAP	RTIAP	Place just below the tibial tuberosity
23、24	LTIAD	RTIAD	Place the marker half way down the lower leg on the crest (palpable 'shin') of the tibia
25、26	LANK	RANK	The lateral malleolus is the distal end of the fibula, located on the lateral ankle
27、28	LHEE	RHEE	Placed on the calcaneus at the same height above the plantar surface of the foot as the toe marker
29、30	LTOE	RTOE	Placed over the second metatarsal head, on the mid-foot side of the equinus break between fore-foot andmid-foot
31、32	LMED	RMED	The medial malleolus is the distal end of the tibia, located on the medial ankle

33、34	LFMH	RFMH	The 1st metatarsal is the hallux
35、36	LSMH	RSMH	Place the center of the marker on the line of the second metatarso-phalangeal joint so that the marker is in the center of the bone (from medial to lateral)
37、38	LVMH	RVMH	The 5th metatarsal is the most lateral of the metatarsal bones. The base of the metatarsal is the most prominent part of the bone

Table S2. Common training configuration used for all models

Item	Setting
Framework	PyTorch
Random seed	42
Optimizer	AdamW
Initial learning rate	Tuned (default 1×10^{-4})
Weight decay	Tuned (default 2×10^{-6})
Learning-rate scheduler	ReduceLROnPlateau
Scheduler patience	12 epochs
Scheduler factor	0.5
Minimum learning rate	1×10^{-6}
Gradient clipping	3
Mixed precision training	Automatic Mixed Precision (AMP)
Batch size	Randomly sampled from {64, 128, 256}
Hyperparameter search epochs	Max 160
Final training epochs	Adaptive (up to 220)
Early stopping	Patience = 35 epochs
Loss function	Weighted Mean Squared Error
Loss weights	Randomly sampled during tuning
Activation	LeakyReLU
LeakyReLU slope	Tuned (0.05–0.20)
Output activation	Tanh
Hyperparameter search	Random Search (120 trials)

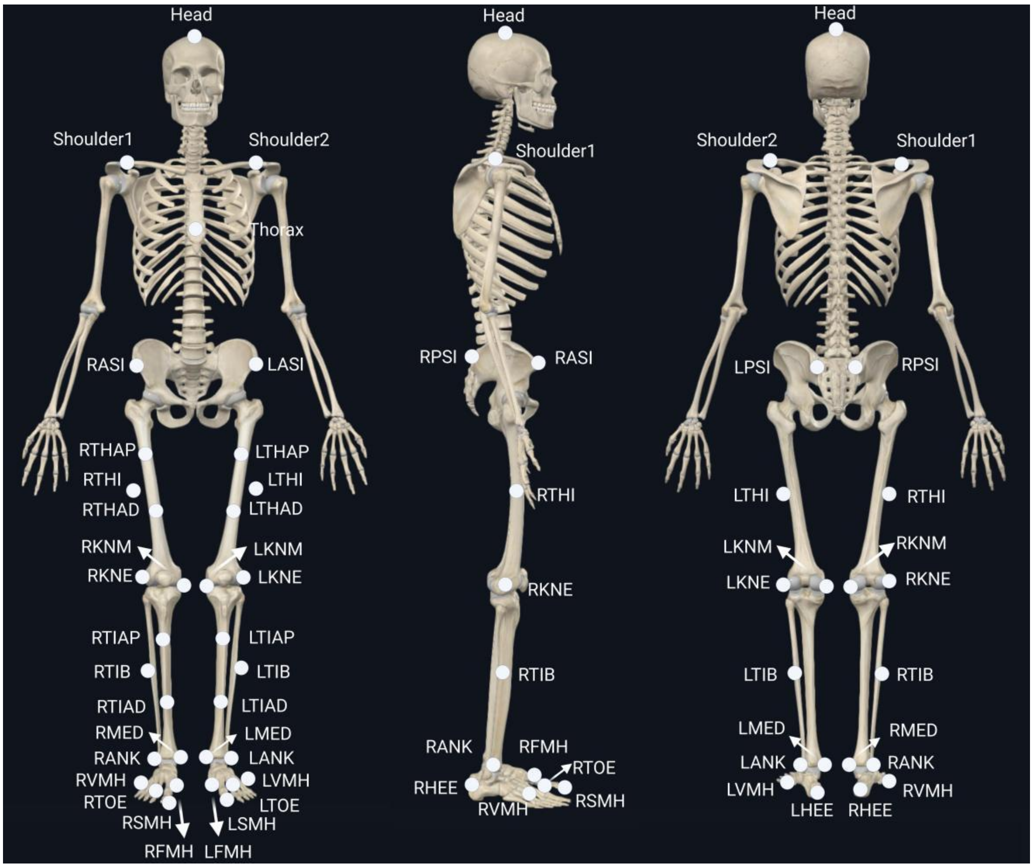

Figure S1.Illustration of marker-set.

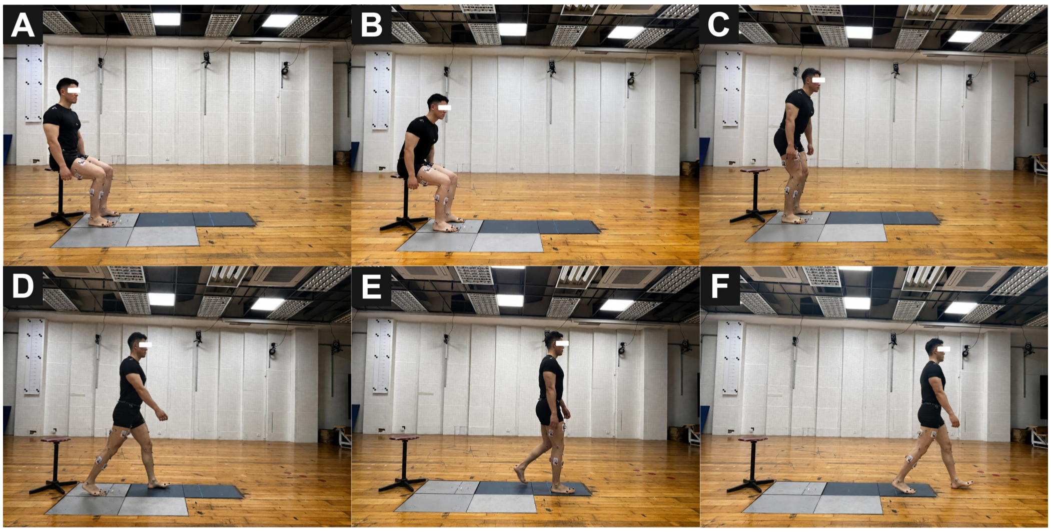

Figure S2. Representative sequence of the sit-to-walk task.

Panels A–F show a representative sequence of the sit-to-walk task from quiet sitting to standing and subsequent gait initiation. Participants began from a standardized seated posture with both feet placed on the first force plate and were instructed to stand up and initiate forward walking after the start command. The participant's eyes were masked to protect identity.

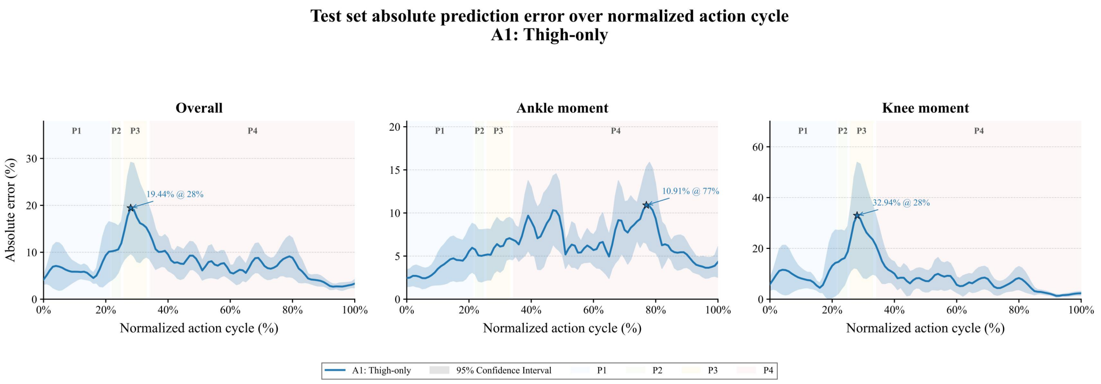

Figure S3. Absolute prediction error of the A1: Thigh-Only model over the normalized action cycle in the test set.

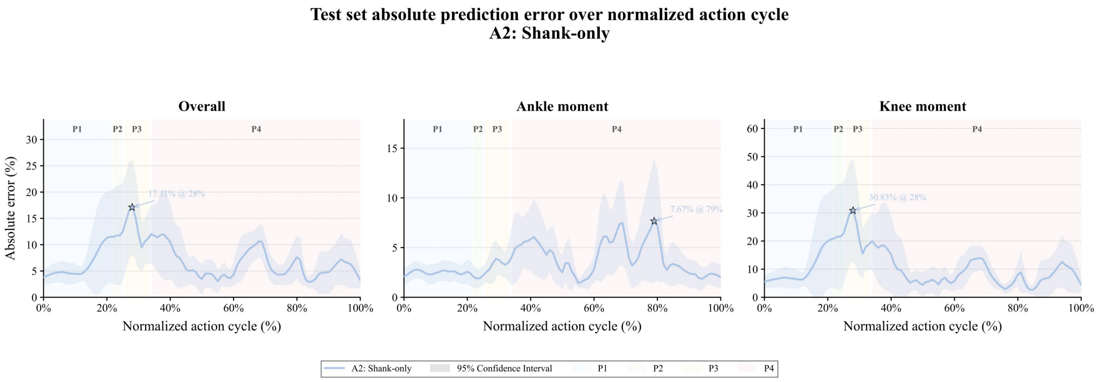

Figure S4. Absolute prediction error of the A2: Shank-Only model over the normalized action cycle in the test set.

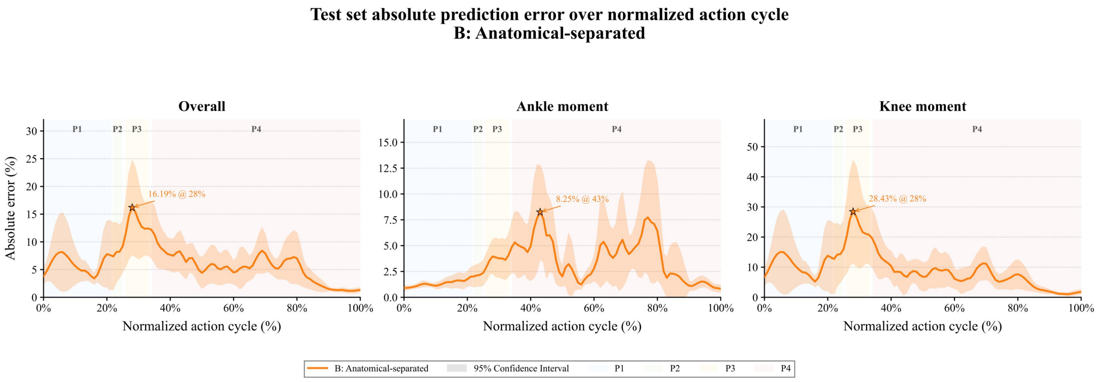

Figure S5. Absolute prediction error of the B: Anatomical-separated model over the normalized action cycle in the test set.

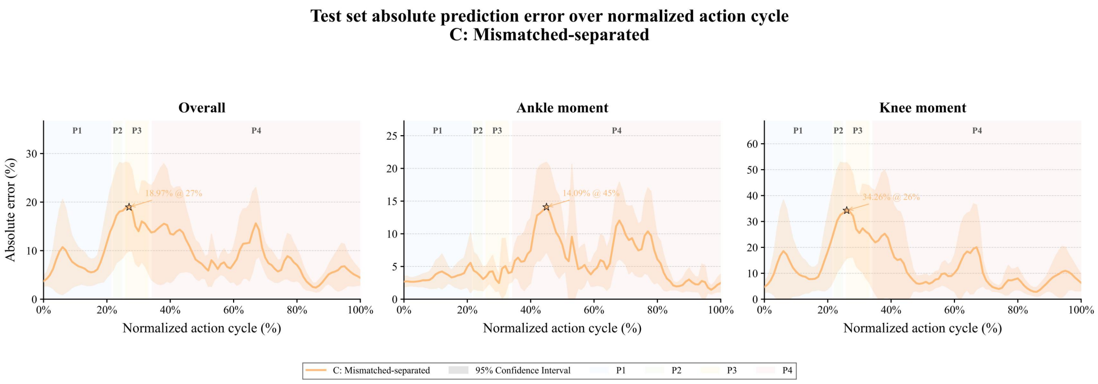

Figure S6. Absolute prediction error of the C: Mismatched-separated model over the normalized action cycle in the test set.

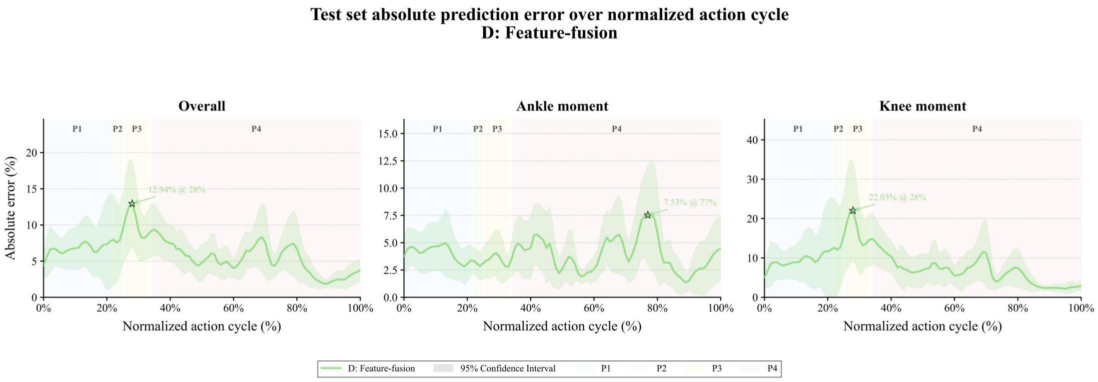

Figure S7. Absolute prediction error of the D: Feature-fusion model over the normalized action cycle in the test set.
